# Supplementary material for: White matter integrity in mice requires continuous myelin synthesis at the inner tongue
Source: Nat Commun. 2022 Mar 4;13:1163. doi: 10.1038/s41467-022-28720-y (PMC8897471; doi:10.1038/s41467-022-28720-y)

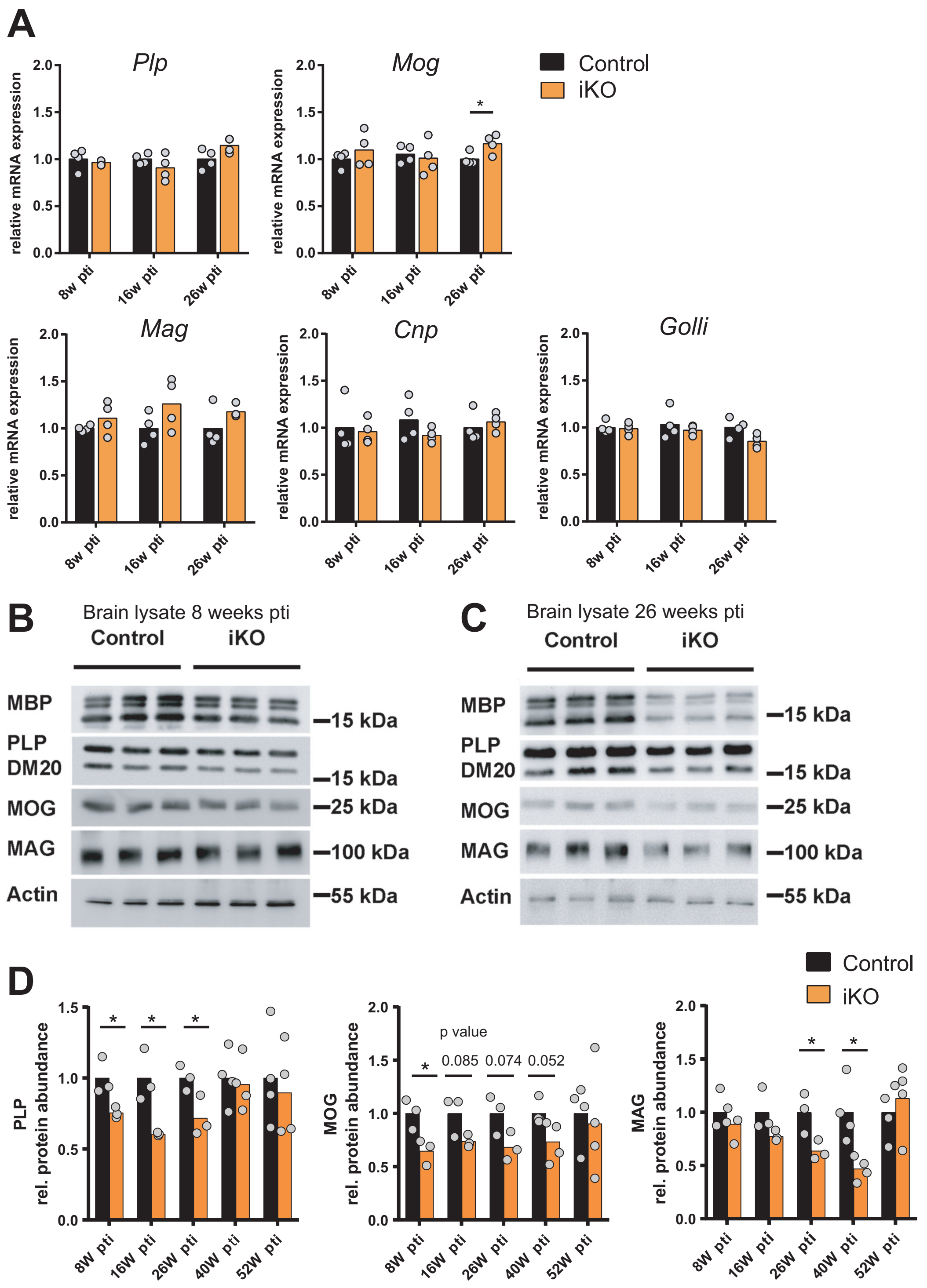

Figure S1 (related to Fig 1D-F): Quantification of mRNA abundance of myelin genes and myelin proteins by immunoblotting at indicated time points after ablation of *Mbp*.
(A) qRT-PCR of brain lysate showed that mRNA levels of indicated myelin genes are unchanged after ablation of *Mbp*. (n=4, male mice) two-tailed unpaired t-test, control vs iKO: *Mog* expression at 26 weeks pti: p=0.04 (p < 0.05 (*)). **(B)** Immunoblot of brain lysate 8 weeks pti and **(C)** 26 weeks pti and quantitation in **(D)**. The protein abundance of PLP is reduced, for MOG and MAG trends towards decreased abundance is detectable: PLP 8 weeks pti (74%), 16 weeks pti (60%), 26 weeks pti (71%); MOG 8 weeks pti (64%), 16 weeks pti (73%), 26 weeks pti (68%); MAG 8 weeks pti (88%), 16 weeks pti (73%), 26 weeks pti (63%) Single data points in the graphs represent individual mice (n-number, female and male mice). (multiple t-test, p<0.05 (*), exact p-values are provided in the Source data file). For *Mbp* mRNA abundance and MBP protein levels in brain lysate see Fig 1D and E and Fig. 1F and G respectively. Source data are provided as a Source Data file.

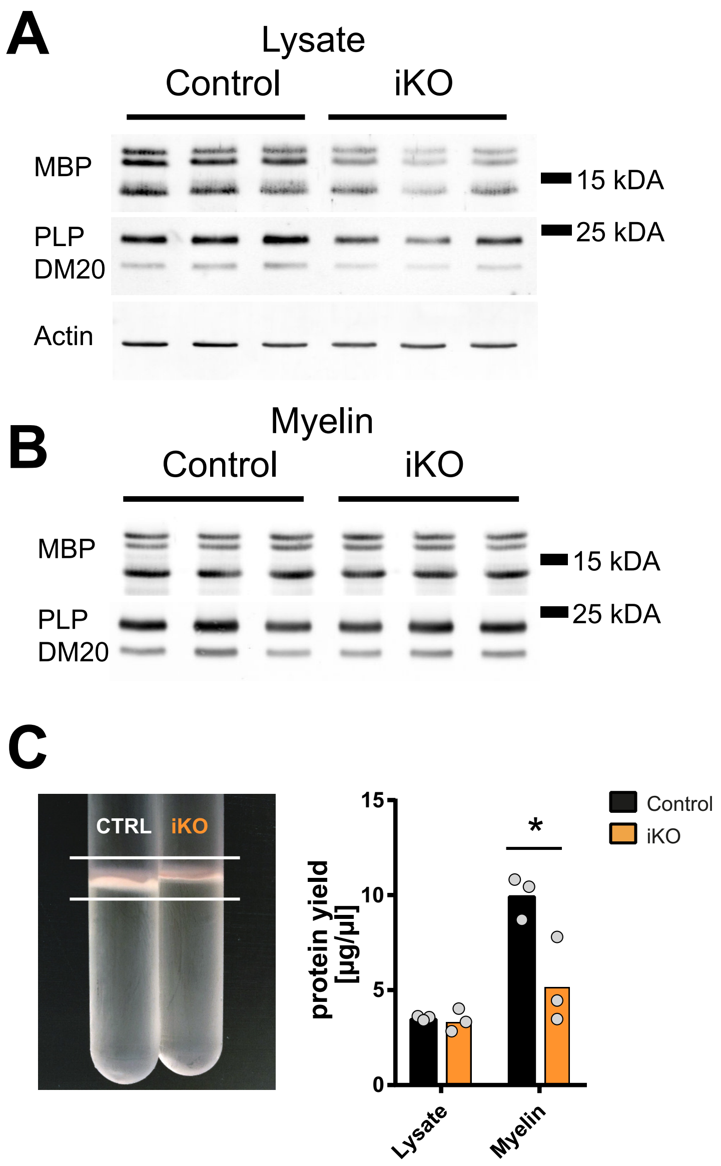

Figure S2 (related to Fig. 1G): Abundance of MBP in biochemically purified myelin.
(A) Immunoblot of MBP and PLP in Lysate and **(B)** myelin biochemically purified from brains reveals a reduction on lysate level but no changes in MBP and PLP protein abundance in the myelin fraction. **(C)** Isolation of a myelin enriched fraction shows a visibly reduced yield of myelin membrane and protein from iKO brains 26 weeks pti, Bradford protein assay of lysate and myelin (two-tailed unpaired t-test; control vs iKO (n=3) lysate: p=0.6983; purified myelin: p=0.0315; p <0 .05 (*)) Single data points in the graphs represent individual mice (n-number, male mice). Source data are provided as a Source Data file.

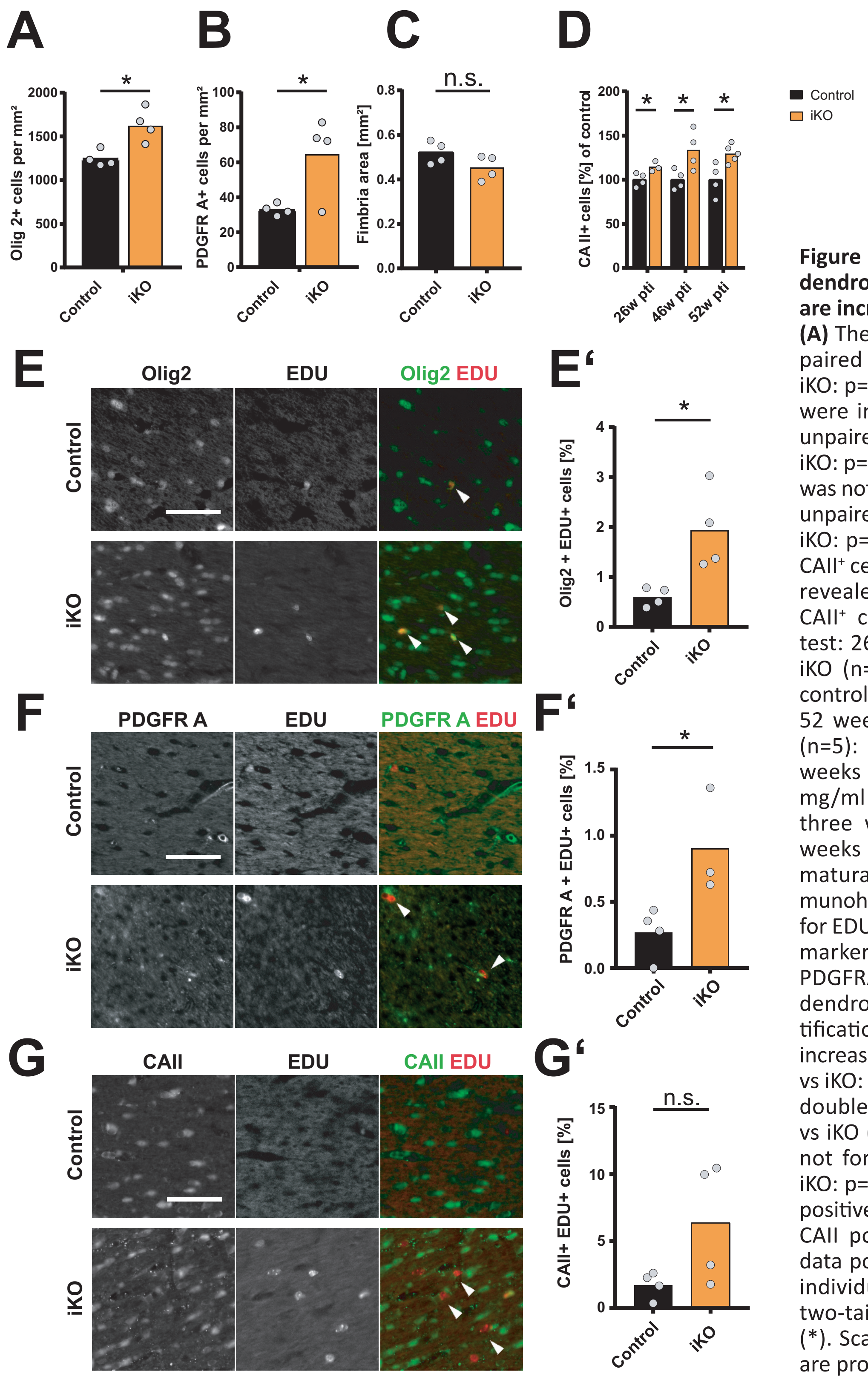

Figure S3 (related to Fig. 1): Oligodendrocyte numbers in the fimbria are increased after *Mbp* ablation. **(A)** The densities of Olig2$^+$ (n=4, unpaired two-tailed t-test: control vs iKO: p=0.018) and **(B)** PDGFRA$^+$ cells were increased 46 weeks pti (n=4, unpaired two-tailed t-test: control vs iKO: p=0.048) while the fimbria area was not significantly decreased (n=4, unpaired two-tailed t-test: control vs iKO: p=0.126) **(C)**. Quantification of CAII$^+$ cells at 26, 46 and 52 weeks pti revealed a consistent increase in CAII$^+$ cells (unpaired two-tailed t-test: 26 weeks pti, control (n=4) vs iKO (n=3): p=0.034; 40 weeks pti, control (n=4) vs iKO (n=4): p=0.048; 52 weeks pti, control (n=4) vs iKO (n=5): p=0.043) **(D)**. Mice at 40 weeks pti were treated with 0.2 mg/ml EdU in drinking water for three weeks followed by a three weeks chase period to allow cell maturation of labeled cells. Immunohistochemistry of cells positive for EDU$^+$ and oligodendrocyte linage marker Olig2 **(E)**, the OPC marker PDGFRA **(F)** and the mature oligodendrocyte marker CAII (**G**). Quantification showed a significant increase in EDU$^+$ Olig 2$^+$ (n=4, control vs iKO: p=0.043) and EDU$^+$ PDGFR A$^+$ double positive cells (control (n=4) vs iKO (n=3): p=0.0355) **(E', F')**, but not for EDU$^+$ CAII$^+$ (n=4, control vs iKO: p=0.1295). **(E', F', G')** % of EDU positive cells from Olig 2, PDGFRA or CAII positive cells per FOV, Single data points in the graphs represent individual male mice (n-number), two-tailed unpaired *t*-test; $p < 0.05$ (*). Scale bars: 50 µm. Source data are provided as a Source Data file.

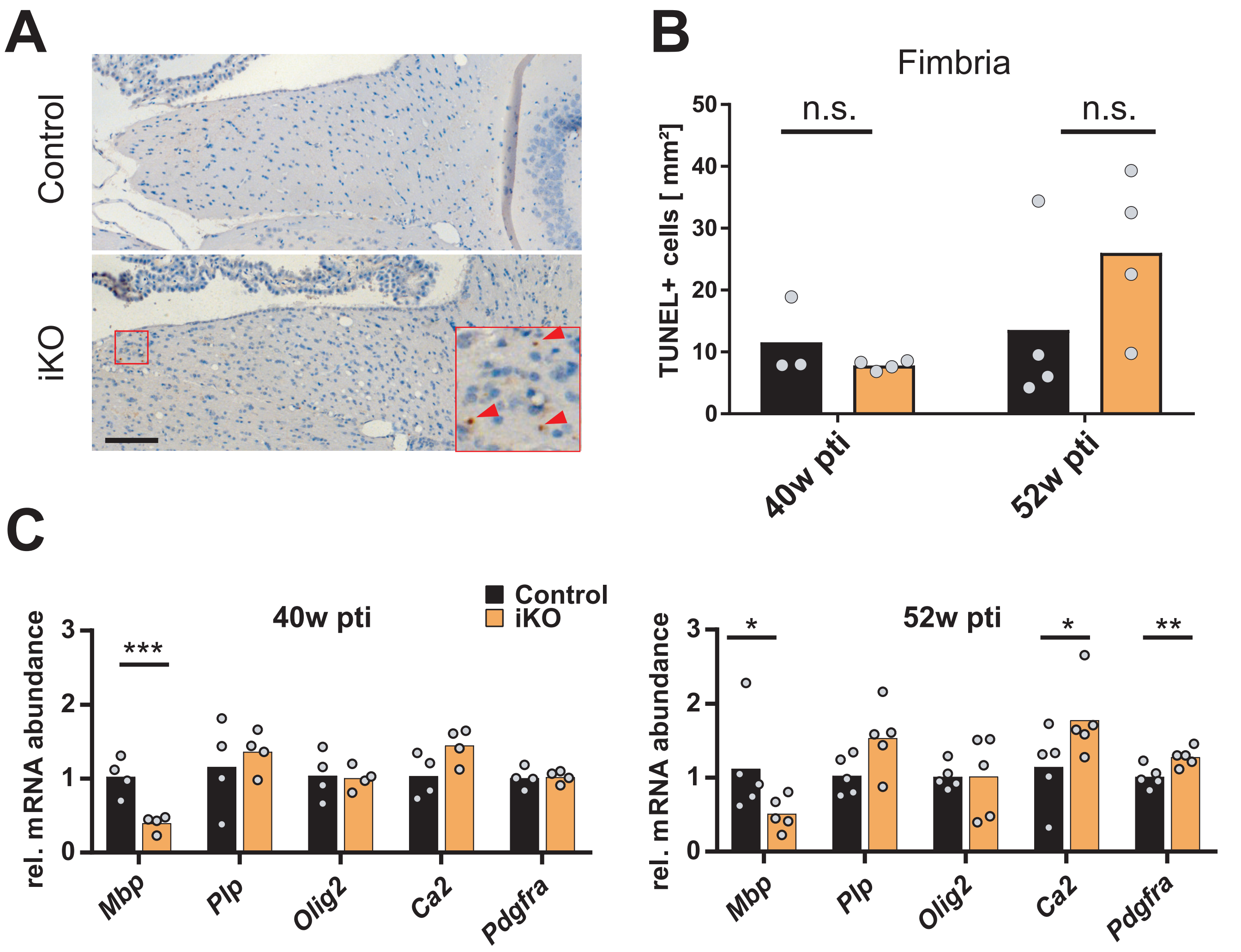

Figure S4 (related to Fig. 1): Survival of recombined oligodendrocytes.
(A) TUNEL cell death assay to test for increased cell death in the fimbria of iKO animals showed no significant increase in TUNEL-positive cells at 40 (male mice) and 52 weeks pti (female mice) depicted in **(B)**. Chromogenic labeling, 2 fimbria per animal, 40 weeks pti: control (n=3), iKO (n=4) two-tailed unpaired *t*-test, scale bar 100 µm. **(C)** qRT-PCR analysis of corpus callosum at 40 and 52 weeks pti. Expression of *Mbp* mRNA is reduced to 50% of control (40 weeks pti (n=4, male mice): p=0.005; 52 weeks pti (n=5, female mice): p=0.038) while the expression of *Plp* and *Olig 2* is unchanged. The OPC marker *Pdgfra* (control vs iKO (n=5): p=0.04) and the mature oligodendrocyte marker *Ca2* (control vs iKO (n=5): p=0.0035) show increased expression at 52 weeks pti. Single data points in the graphs represent individual mice (n-number). Two-tailed unpaired t-test, $p < 0.05$ (*), $p < 0.01$ (**), $p < 0.001$ (***).
Source data are provided as a Source Data file.

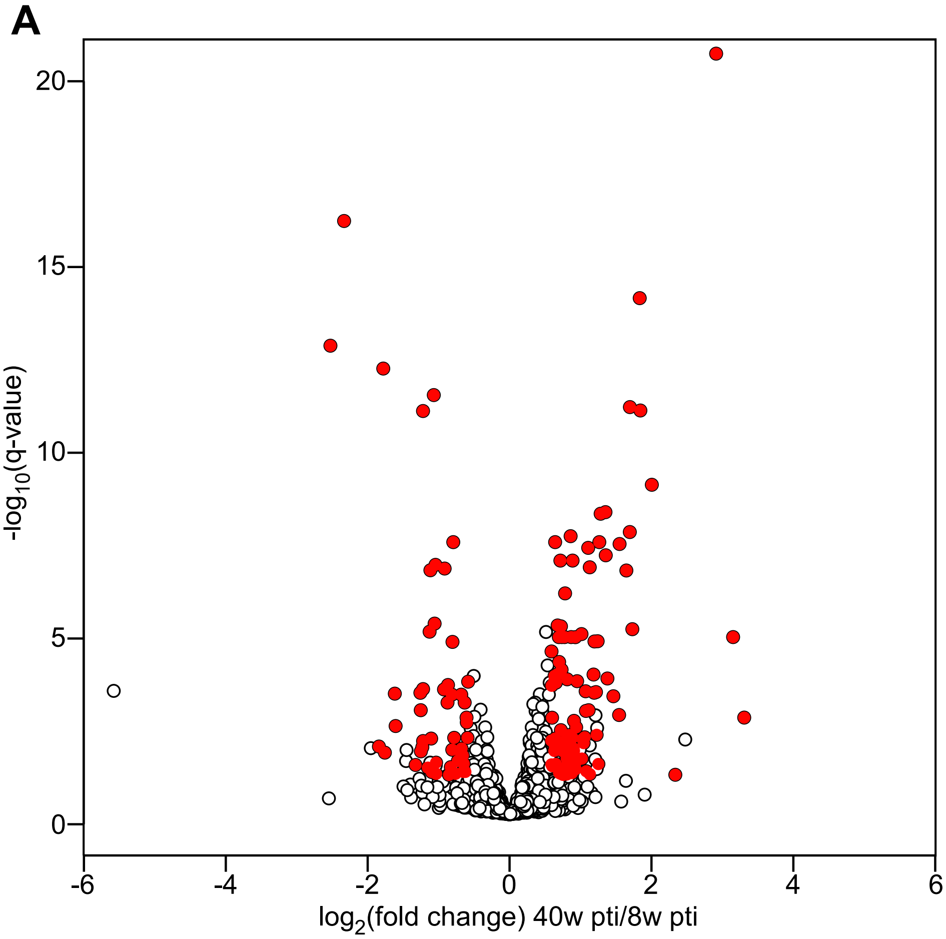

Figure S5 (related to Fig. 1): Proteome alterations in the late stage of MBP deficiency.
(A) Volcano plot visualizing differences in normalized protein abundance between early (iKO/Ctrl 8w) and late (iKO/Ctrl 40w) time points after MBP deletion. From the 1863 proteins in the entire dataset (open black circles), 149 proteins remained after stringent filter criteria: log2 fold-change (FC) >|0.58|, q-value < 0.05, min. 6 out of 8 values detected per group (filled red circles, see also Supplementary Table 1). As no imputation of missing values was performed, proteins exclusive for iKO or Ctrl are not part of the analysis and were considered separately by manual analysis. **(B)** Clustering of the normalized abundance of the 149 proteins derived from the volcano plot in **(A)**. The heatmap guided the selection of categories and their protein representatives for the visualization of pathological events in Fig. 1I-K. Selected proteins are shown in red, with an enlarged protein acronym (nomenclature as used in Fig. 1H-K) shown adjacent to the protein ID column of the heatmap.
Source data are provided as a Source Data file.

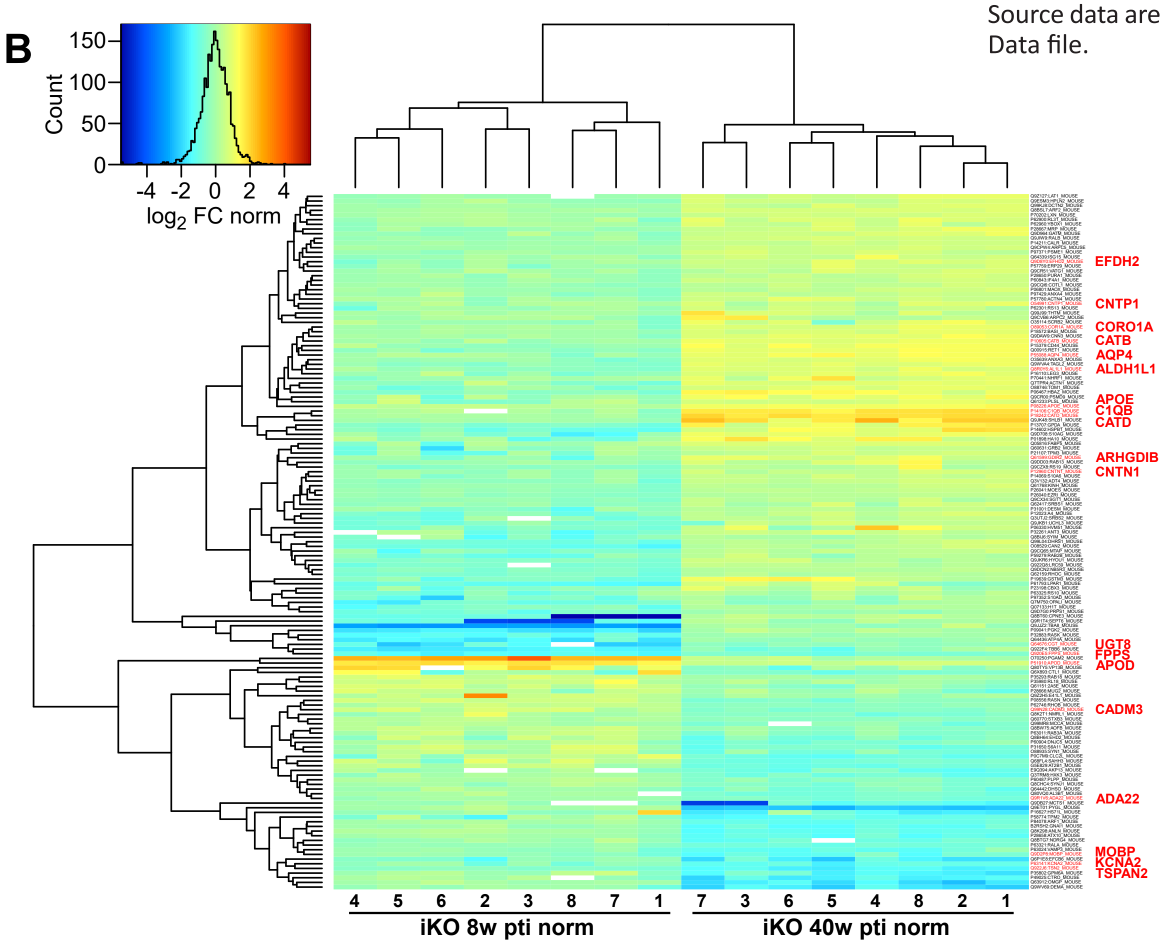

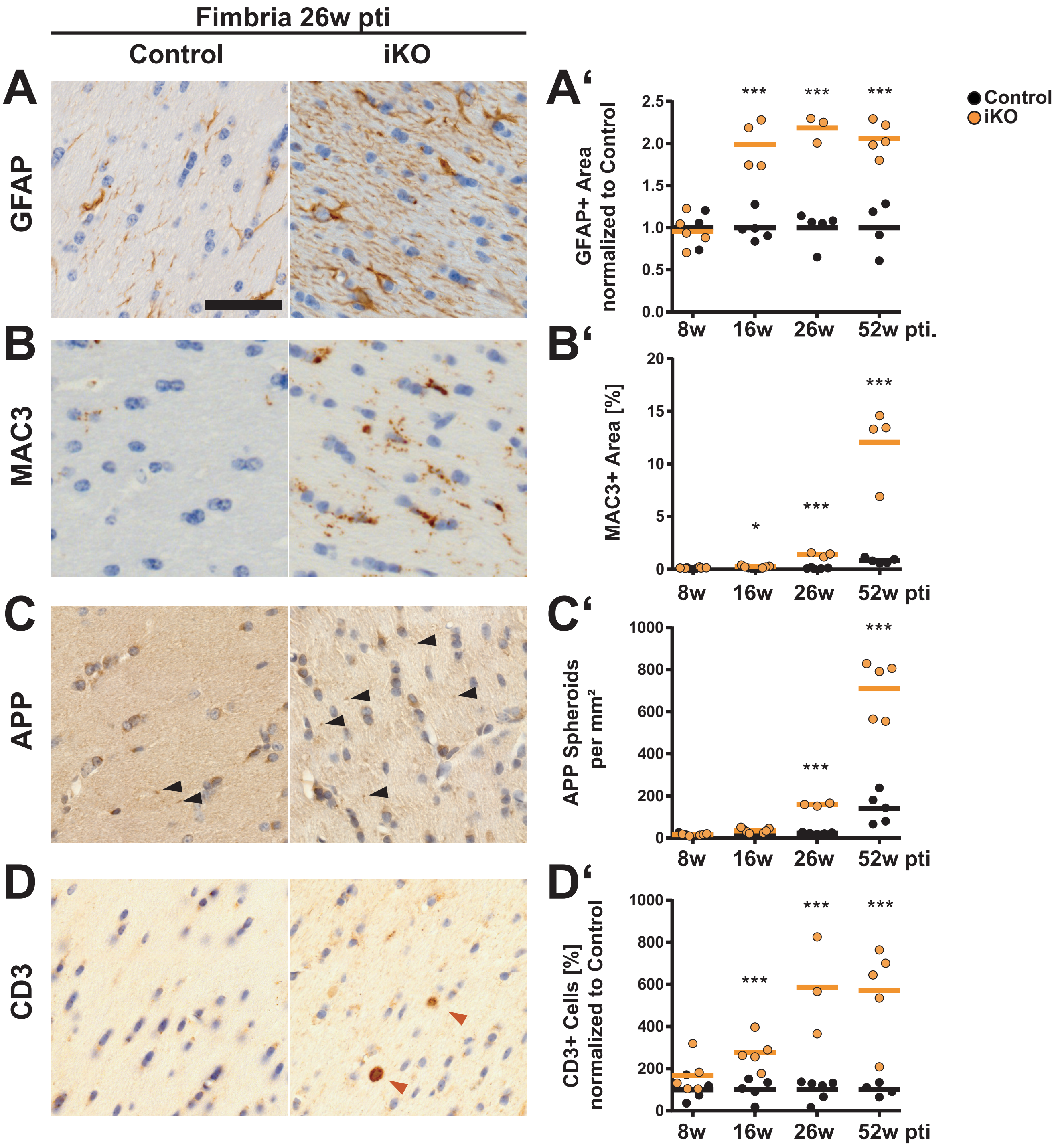

Figure S6 (related to Fig. 1): Slow development of inflammation and neuropathology in the fimbria.
(A-D) Immunhistochemical staining of GFAP, MAC3, APP and CD3 in the fimbria 26 weeks pti and in **(A'-D')** quantification of indicated time points. An increase in GFAP^{+} area **(A)** indicates astrogliosis beginning between 8 and 16 weeks pti (8 weeks pti, control (n=3) vs iKO (n=5): p=0.805; 16 weeks pti, control (n=5) vs iKO (n=4): p=0.00033; 26 weeks pti, control (n=5) vs iKO (n=3): p=0.000121; 52 weeks pti, control (n=4) vs iKO (n=5): p=0.000366) **(A')**. The increase of MAC3^{+} area **(B)** indicates microglial activity beginning between 8 and 16 weeks pti and increasing until 52 weeks pti (8 weeks pti, control (n=3) vs iKO (n=5): p=0.223; 16 weeks pti, control (n=4) vs iKO (n=5): p=0.0179; 26 weeks pti, control (n=5) vs iKO (n=3): p<0.0001; 52 weeks pti, control (n=5) vs iKO (n=4): p=0.00016) **(B')**. Axonal pathology observed by labeling of APP spheroids **(C)** appears late at 26 weeks pti and increases until 52 weeks pti (8 weeks pti, control (n=4) vs iKO (n=5): p=0.772; 16 weeks pti, control (n=5) vs iKO (n=6): p=0.078; 26 weeks pti, control (n=5) vs iKO (n=3): p<0.0001; 52 weeks pti, control (n=5) vs iKO (n=5): p<0.0001) **(C')**. Increased numbers of T-cells **(D)** can be observed already at 16 weeks pti indicating immune response (cells/mm^2 normalized to control: 8 weeks pti, control (n=4) vs iKO (n=5): p=0.234; 16 weeks pti, control (n=5) vs iKO (n=5): p=0.0031; 26 weeks pti, control (n=6) vs iKO (n=3): p=0.0011; 52 weeks pti, control (n=4) vs iKO (n=5): p=0.004 **(D')**. Analysis of 2 fimbria (GFAP, MAC3, APP) or 6 (CD3) fimbria per animal. Single data points in the graphs represent individual mice (n-number) For the time points 8, 16 and 26 weeks pti male mice were used, for the time point 52 weeks pti female mice. Two-tailed unpaired *t*-test; $p < 0.05$ (*), $p < 0.01$ (**), $p < 0.001$ (***). Scale bars: 50 µm
Source data are provided as a Source Data file.

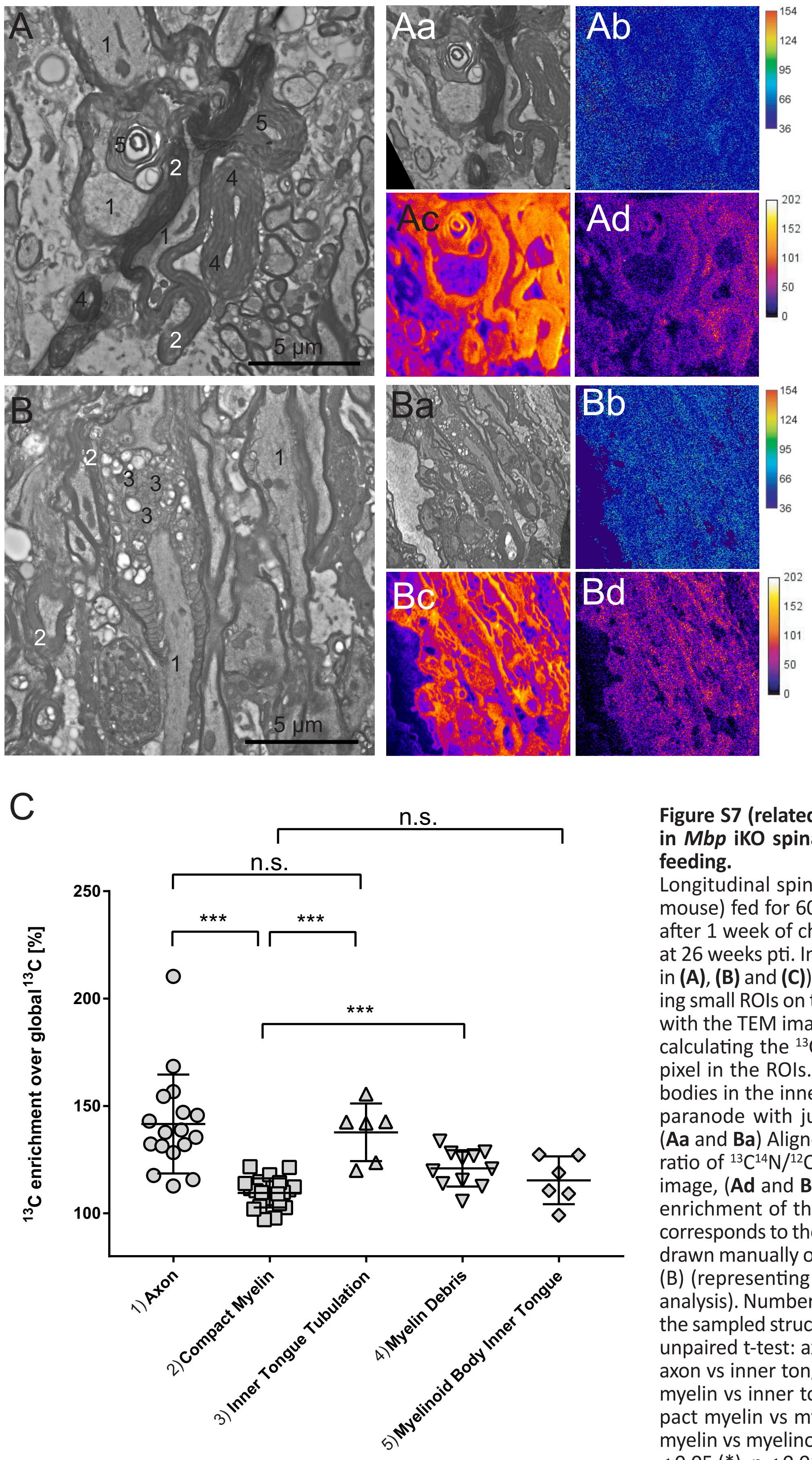

Figure S7 (related to Figure 2): NanoSIMS imaging in *Mbp* iKO spinal cord after 60 d ^{13}C-lysine diet feeding.

Longitudinal spinal cord TEM section of iKO (male mouse) fed for 60 d with ^{13}C-Lys diet and sacrificed after 1 week of chase with non-labeled control diet at 26 weeks pti. Indicated structures (numbered 1-5 in **(A)**, **(B)** and **(C)**) were analyzed by manually selecting small ROIs on the NanoSIMS image in correlation with the TEM image of the same sampled area, and calculating the $^{13}C^{14}N/^{12}C^{14}N$ isotopic ratio of every pixel in the ROIs. **(A)** Myelin outfolding, myelinoid bodies in the inner tongue and myelin debris. **(B)** A paranode with juxtaparanodal myelin tubulation. (**Aa** and **Ba**) Aligned TEM image, (**Ab** and **Bb**) Image ratio of $^{13}C^{14}N/^{12}C^{14}N$, (**Ac** and **Bc**) $^{12}C^{14}N$ NanoSIMS image, (**Ad** and **Bd**) $^{13}C^{14}N$ NanoSIMS image. **(C)** ^{13}C enrichment of the sampled ROIs. Every data point corresponds to the average value of the sampled ROI drawn manually on the analyzed structure in (A) and (B) (representing the n-number used for statistical analysis). Numbers on the TEM image correspond to the sampled structures in **C**. (mean +/- SD, two-tailed unpaired t-test: axon vs compact myelin: $p<0.0001$; axon vs inner tongue tubulation: $p=0.702$; compact myelin vs inner tongue tubulation: $p<0.0001$; compact myelin vs myelin debris: $p=0.00024$; compact myelin vs myelinoid body inner tongue: $p=0.119$; ($p < 0.05$ (*), $p < 0.01$ (**), $p < 0.001$ (***)). Scale bars: (A and B) 5 µm

Source data are provided as a Source Data file.

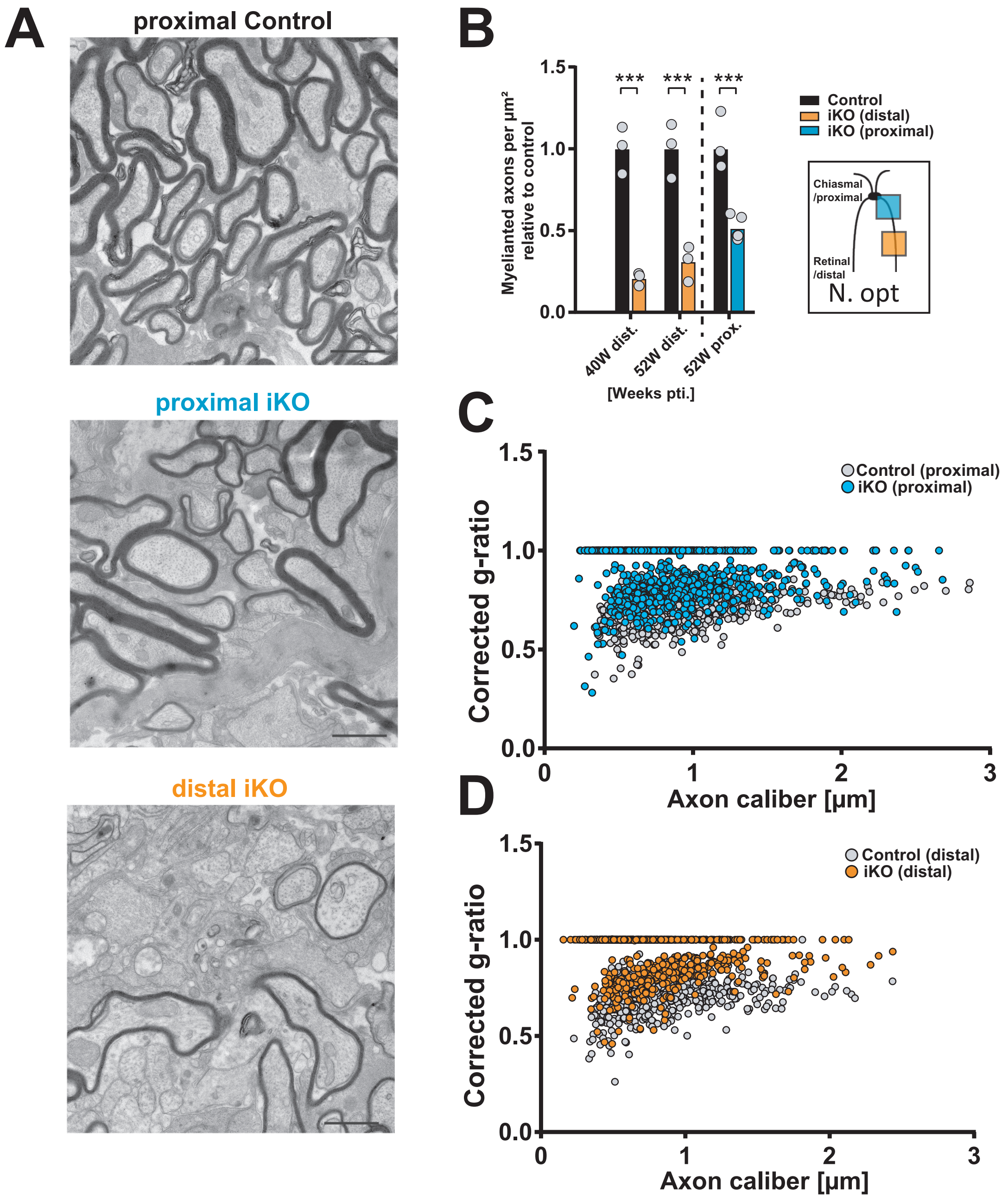

Figure S8 (related to Figure 4): Regional differences in myelination in the optic nerve 52 weeks pti.
(A) TEM image of proximal optic nerve in control and iKO compared to distal optic nerve in iKO. **(B)** Between 40 weeks and 52 weeks pti an increase in the number of myelinated axons is detected indicating remyelination. Single data points in the graphs represent individual male mice (n-number), two-tailed unpaired t-test; 40 weeks pti, distal optic nerve (n=3): p=0.00079; 52 weeks pti, distal optic nerve, control (n=3) vs iKO (n=4): p=0.00081; 52 weeks pti, proximal optic nerve, control (n=4) vs iKO (n=5) : p=0.00045; p<0.001 (***). **(C)** and **(D)** G-ratio measurement in the proximal and distal part of the optic nerve in iKO and control at 52 weeks pti shows thinner myelin sheaths and a large number of unmyelinated axons in the iKO in both regions. N=4-5 animals with analysis of >150 axons per animal. Scale bar: 1 µm
Source data are provided as a Source Data file.

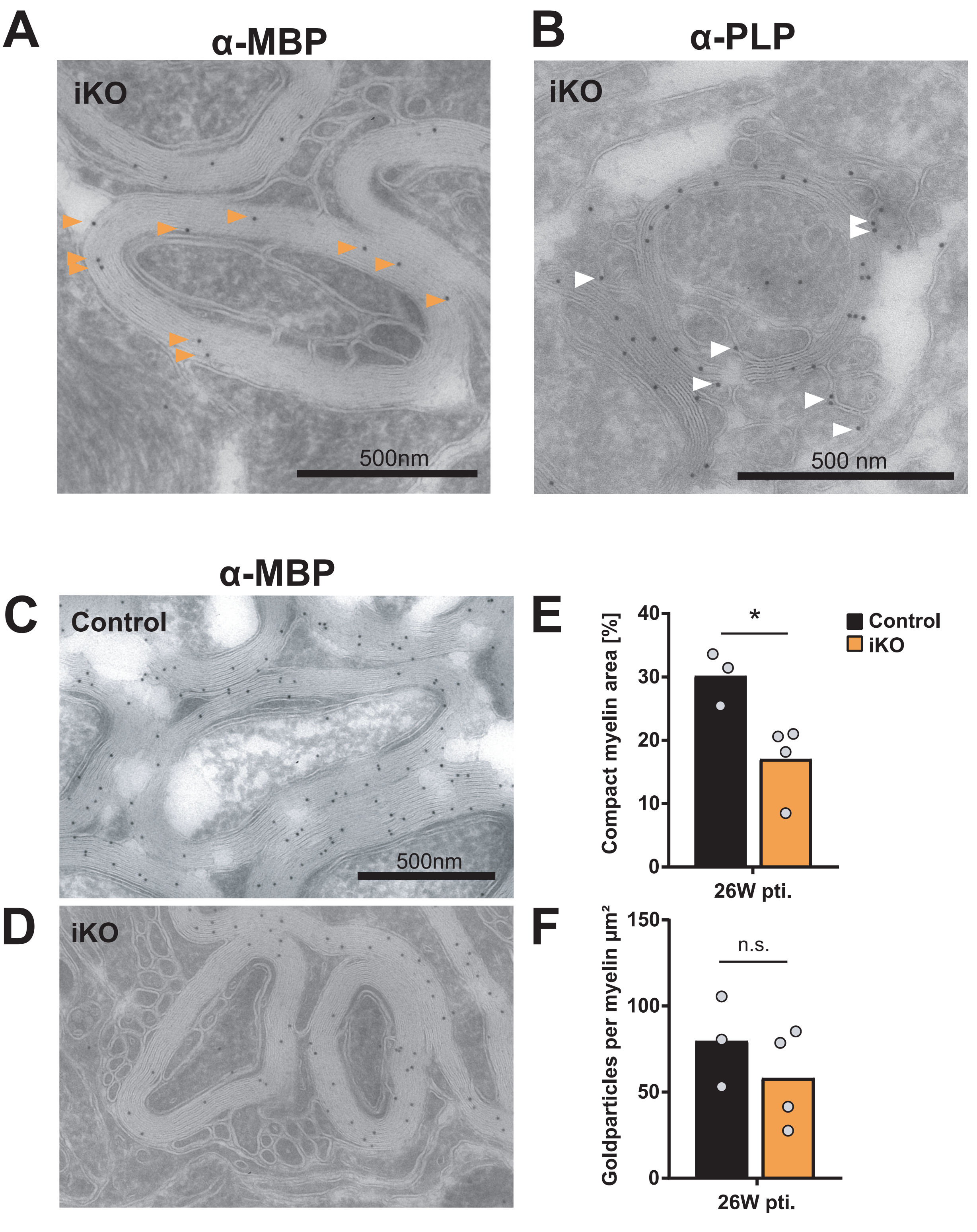

Figure S9 (related to Fig. 3): Membrane tubules are oligodendrocytic membranes that are devoid of MBP.
(A-B) Membrane tubules found at the inner tongue and adjacent to axons are MBP negative **(A)** but show PLP-labeling **(B)** indicating an oligodendrocytic origin. Optic nerve 26 weeks pti, 10 nm protein-A gold particles bound to primary antibody against PLP and MBP. This observation was made in two independent groups of male mice (n=2 controls; n=4 iKO and n=3 wt; n=4 iKO) **(C-D)** The labeling density of MBP in control myelin **(C)** is comparable to iKO myelin **(D)** suggesting little reduction in MBP content within the compact myelin of iKOs. **(E)** The section area occupied by myelin is decreased after *Mbp* iKO. Two-tailed unpaired t-test, control (n=3) vs iKO (n=4): p=0.0438 **(F)** Quantification of 10 nm protein-A-gold particles. At least 4 sections with in total 300 µm² per animal were quantified using a 2 µm grid to randomly select axons, two-tailed unpaired t-test, control (n=3) vs iKO (n=4): p=0.35. Single data points in the graphs represent individual mice (n-number), $p < 0.05$ (*). Scale bar: 500 nm
Source data are provided as a Source Data file.

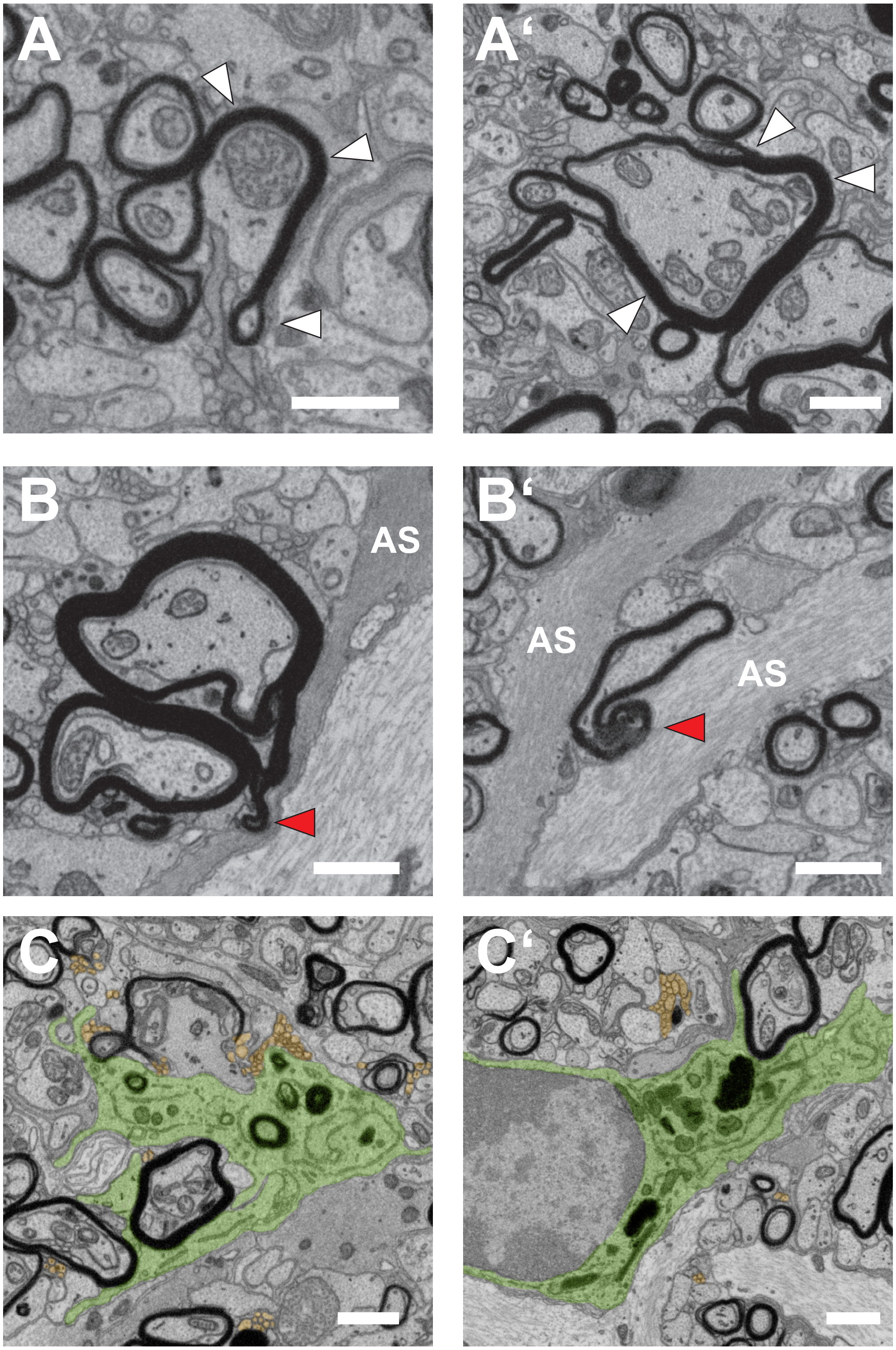

Figure S10 (related to Fig. 7): Myelin outfoldings engulfed by astrocytes and myelin debris incorporated by microglia. (A, A') Myelin outfoldings (white arrow head) are visible in the optic nerve 26 weeks pti. **(B-B')** Astrocytes (AS) are found in direct contact to myelin protrusions (red arrow head). **(C-C')** Microglia (false colored in green) are found close to demyelinating axons (asterisk) and non-compact myelin membrane (false colored in orange). Presence of dark degradative compartments containing myelin debris indicate microglial uptake of myelin. Images were extracted from a FIB-SEM image stack of optic nerve 26 weeks pti. Similar observations were made in two groups of female and male mice (females: n=7 controls; n=10 iKO and n=3 controls; n=5 iKO), (males: n=3 controls; n=5 iKO and n=2 controls; n=4 iKO). Scale bars: 1 µm.

Source data Westernblots

Supplementary Figure 1B: Brain lysate 8 weeks pti

MBP

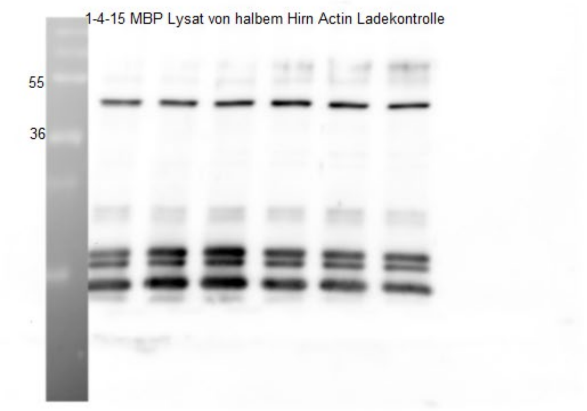

PLP

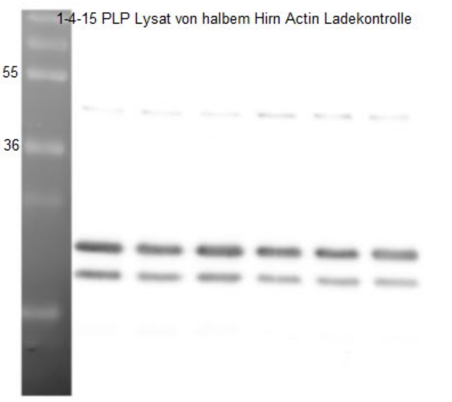

Shutter Time: 30 sec
Binning: 1x1
Scan Mode: increment
Scan Number: 11. scan
Scan Date: 2015-04-01 12:34:32

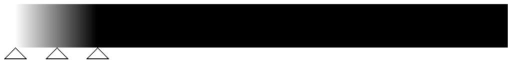

Palette: inverted Grayscale
Range: 3459 - 13774, Gamma: 1.000

MOG

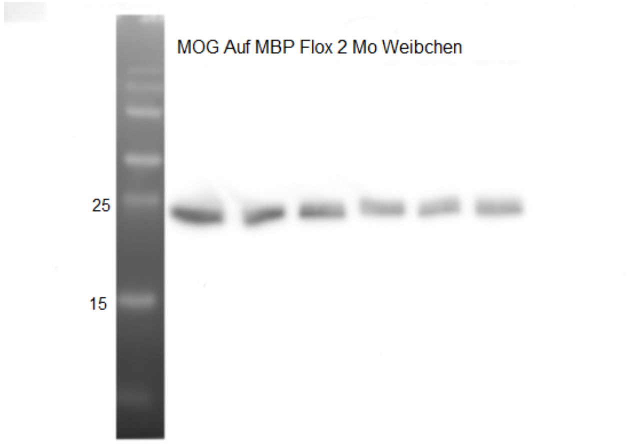

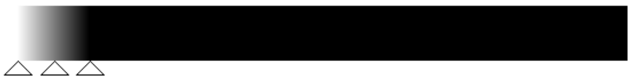

Palette: inverted Grayscale
Range: 4814 - 11972, Gamma: 1.000

MAG

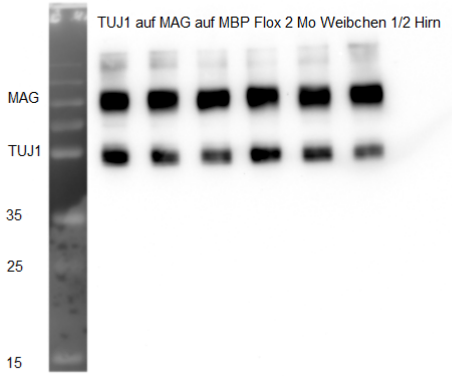

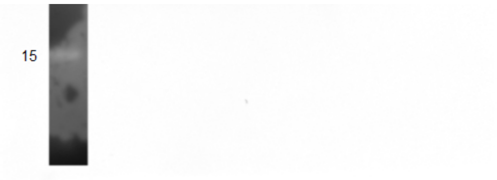

Shutter Time: 30 sec
Binning: 1x1
Scan Mode: increment
Scan Number: 24. scan
Scan Date: 2015-04-16 13:58:50

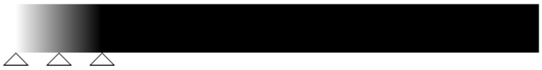

Palette: inverted Grayscale
Range: 6099 - 15878, Gamma: 1.000

Actin loading control on MBP blot

Actin Ladekontrolle auf MBP (15%) Gewebe: MBP FLX vom 16.04.15 2 Monate

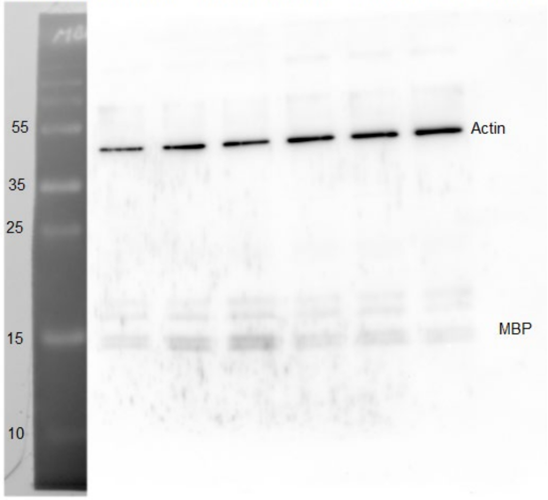

Shutter Time: 30 sec
Binning: 1x1
Scan Mode: increment
Scan Number: 35. scan
Scan Date: 2015-04-20 15:33:44

Supplementary Figure 1C: Brain lysate 26 weeks pti

MBP(Actin) PLP MAG(TUJ1) MOG,35-35. scan.tif 30-APR-2015

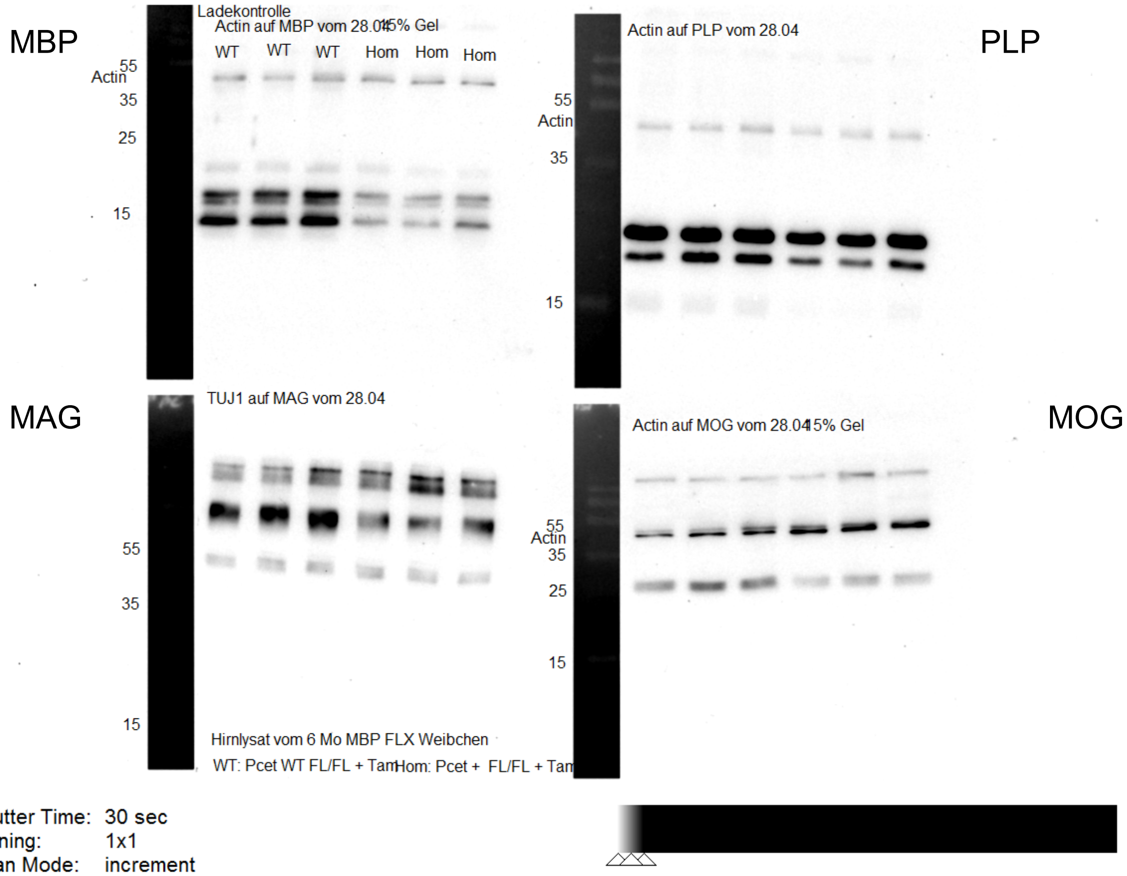

Shutter Time: 30 sec
Binning: 1x1
Scan Mode: increment
Scan Number: 35. scan
Scan Date: 2015-04-30 13:14:55

Palette: inverted Grayscale
Range: 8422 - 11397, Gamma: 1.000

Source data Westernblots

Supplementary Figure 2A and B: 26 weeks pti

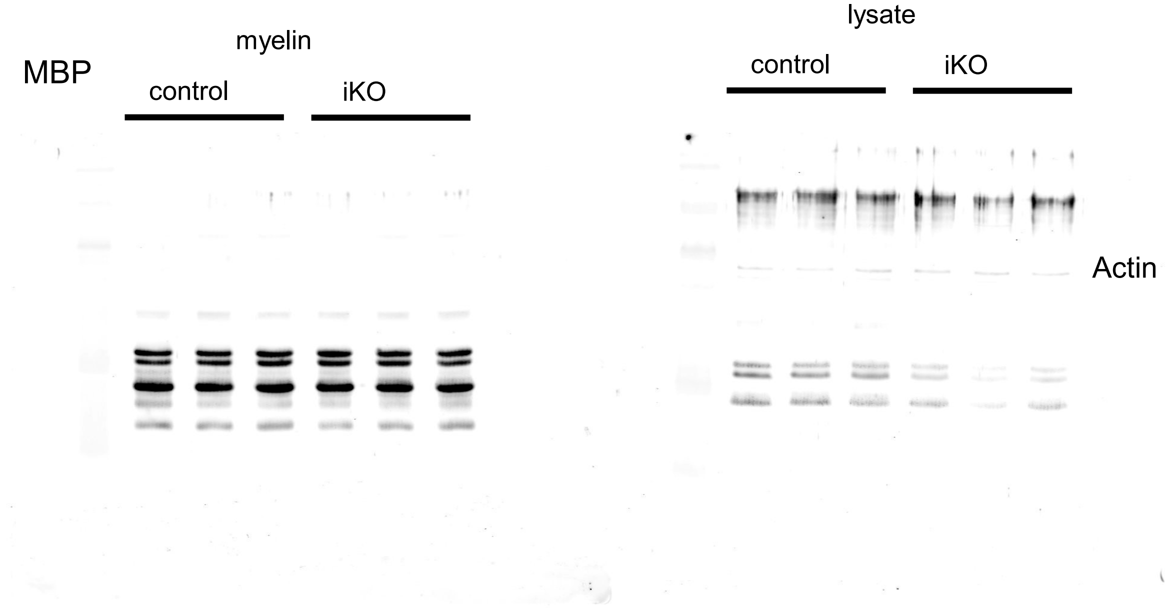

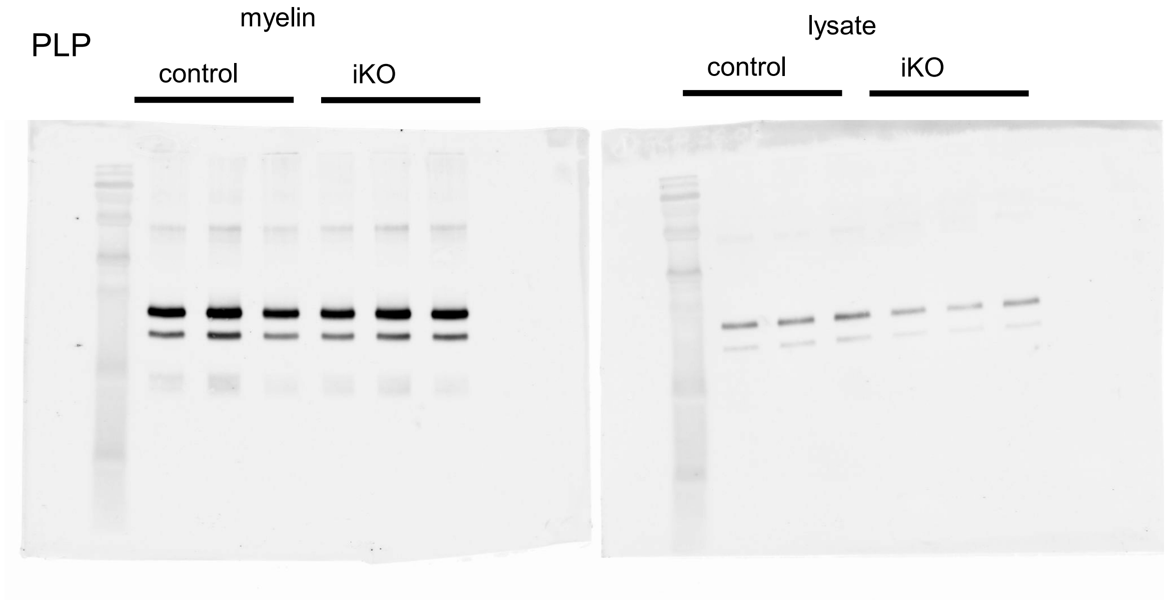

Supplement: Supplementary file 1 — Supplementary Information [file 41467_2022_28720_MOESM1_ESM.pdf]
