# Supplementary material for: White matter integrity in mice requires continuous myelin synthesis at the inner tongue
Source: Nat Commun. 2022 Mar 4;13:1163. doi: 10.1038/s41467-022-28720-y (PMC8897471; doi:10.1038/s41467-022-28720-y)
Supplement: Supplementary file 3 — Description of Additional Supplementary Files [file 41467_2022_28720_MOESM3_ESM.docx]

**Description of Additional Supplementary Files**

**Title: Supplementary Movie 1 related to Figure 5: Internode shortening**

Description: FIB-SEM data stack and 3D reconstruction of a myelinated axon showing the pathological phenotype: The residual internode (in red) is short, shiverer-like membranes processes occur at the juxtaparanode and also emerge at the paranode and bend away from the axon (indicated in yellow). The image stack was recorded with a voxel size of 5 nm x 5 nm x 50 nm; the movie consists of 747 images cropped from the original data stack. The optic nerve (male mouse) was fixed chemically and embedded using the reduced osmium-thiocarbohydrazide-osmium (rOTO) method (Deerinck et al., 2010). The myelinated axon was reconstructed using IMOD (Kremer et al., 1996).

**Title: Supplementary Movie 2 related to Figure 6: Myelin tubulation at the juxtaparanode**

Description: FIB-SEM data stack with indicated phenotype of myelin tubulation occurring at the juxtaparanode. The data stack was recorded with a voxel size of 5 nm x 5 nm x 50 nm. The movie consists of 318 images which were colored using IMOD. The optic nerve sample was prepared by high-pressure freezing and freeze substitution (HPF/FS) at 16 weeks pti (male mouse).

**Title: Supplementary Movie 3: Axonal wrapping in shiverer optic nerve**

Description: FIB-SEM data stack of an optic nerve sample of a male shiverer mouse at 8 weeks of age. Coloration indicates an axon and the associated shiverer membrane wrapping. The data stack was recorded with a voxel size of 5 nm x 5 nm x 50 nm. The sample was prepared by chemical fixation and rOTO embedding, coloration was performed in IMOD.

**Title: Supplementary Movie 4 related to Figure 7: Myelin thinning and formation of myelinoid bodies**

Description: FIB-SEM data stack showing a myelinated axon 26 weeks pti (male mouse). Substantial myelin thinning and the formation of myelinoid bodies are visible. The sample was prepared by chemical fixation and rOTO embedding. Outline of myelin (in yellow) and axon (in red) and myelinoid bodies in different colors. The images were recorded with a voxel size of with 5 nm x 5 nm x 50nm, the movie consists of 404 images cropped from the original data stack (same as used for Supplementary Movie 1). Coloration was performed in IMOD.

**Title:** Supplementary Data 1

Description: Proteomic data set with identification and quantification of detected proteins in all analyzed conditions
