# Supplementary material for: White matter integrity in mice requires continuous myelin synthesis at the inner tongue
Source: Nat Commun. 2022 Mar 4;13:1163. doi: 10.1038/s41467-022-28720-y (PMC8897471; doi:10.1038/s41467-022-28720-y)
Supplement: Supplementary file 9 — Reporting Summary [file 41467_2022_28720_MOESM9_ESM.pdf]

nature research

Corresponding author(s): Dr. Wiebke Möbius

Last updated by author(s): Jan 4, 2022

Reporting Summary

Nature Research wishes to improve the reproducibility of the work that we publish. This form provides structure for consistency and transparency in reporting. For further information on Nature Research policies, see our Editorial Policies and the Editorial Policy Checklist.

Statistics

For all statistical analyses, confirm that the following items are present in the figure legend, table legend, main text, or Methods section.

n/a	Confirmed	
☐	☒	The exact sample size (*n*) for each experimental group/condition, given as a discrete number and unit of measurement
☐	☒	A statement on whether measurements were taken from distinct samples or whether the same sample was measured repeatedly
☐	☒	The statistical test(s) used AND whether they are one- or two-sided<br>*Only common tests should be described solely by name; describe more complex techniques in the Methods section.*
☒	☐	A description of all covariates tested
☒	☐	A description of any assumptions or corrections, such as tests of normality and adjustment for multiple comparisons
☐	☒	A full description of the statistical parameters including central tendency (e.g. means) or other basic estimates (e.g. regression coefficient) AND variation (e.g. standard deviation) or associated estimates of uncertainty (e.g. confidence intervals)
☐	☒	For null hypothesis testing, the test statistic (e.g. *F*, *t*, *r*) with confidence intervals, effect sizes, degrees of freedom and *P* value noted<br>*Give P values as exact values whenever suitable.*
☒	☐	For Bayesian analysis, information on the choice of priors and Markov chain Monte Carlo settings
☒	☐	For hierarchical and complex designs, identification of the appropriate level for tests and full reporting of outcomes
☒	☐	Estimates of effect sizes (e.g. Cohen's *d*, Pearson's *r*), indicating how they were calculated

Our web collection on statistics for biologists contains articles on many of the points above.

Software and code

Policy information about availability of computer code

Data collection	Microscopy: ZEN 2012 blue edition, Carl Zeiss Microscopy; ATLAS3D Vers. 5.2, Fibics, Carl Zeiss Microscopy, LasAF Vers. 3.7.423463, Leica Microsystems<br>Proteomics: Waters MassLynx 4.1; Expression analysis: NIH PrimerBlast (using Primer3web version 4.1.0)
Data analysis	Microscopy Image Browser (RRID:SCR_016560; URL: http://mib.helsinki.fi/)<br>IMOD (RRID:SCR_003297; URL: http://bio3d.colorado.edu/imod)<br>Excel 2013<br>GraphPad Prism (v7.0 and 6v) ,https://www.graphpad.com<br>ImageJ (Fiji) (RRID:SCR_002285; URL: http://fiji.sc)<br>Proteomics: Waters ProteinLynx Global Server (PLGS) version 3.0.3, ISOQuant Version 1.6, Bioconductor R packages limma version 3.38.3 and q-value version 2.14.1<br>The Matlab (the Mathworks Inc, Natick, MA) scripts used for analysis of NanoSIMS data can be obtained from Silvio Rizzoli.

For manuscripts utilizing custom algorithms or software that are central to the research but not yet described in published literature, software must be made available to editors and reviewers. We strongly encourage code deposition in a community repository (e.g. GitHub). See the Nature Research guidelines for submitting code & software for further information.

Data

Policy information about availability of data

All manuscripts must include a data availability statement. This statement should provide the following information, where applicable:

- Accession codes, unique identifiers, or web links for publicly available datasets
- A list of figures that have associated raw data
- A description of any restrictions on data availability

The FIB-SEM datasets generated and/or analyzed during the current study are available from the corresponding author upon reasonable request.
The original 3D electron microscopy data (FIB-SEM image stacks) on which the supplementary movies are based, are deposited in the open accessible EMPIAR repository (https://www.ebi.ac.uk/empiar/) with the public accession codes EMPIAR-10906 and EMPIAR-10907. The mass spectrometry proteomics data have been deposited to the ProteomeXchange Consortium via the PRIDE (Perez-Riverol et al., 2019) partner repository with the dataset identifier PXD025180.

Field-specific reporting

Please select the one below that is the best fit for your research. If you are not sure, read the appropriate sections before making your selection.

- [x] Life sciences
- [ ] Behavioural & social sciences
- [ ] Ecological, evolutionary & environmental sciences

For a reference copy of the document with all sections, see nature.com/documents/nr-reporting-summary-flat.pdf

Life sciences study design

All studies must disclose on these points even when the disclosure is negative.

Sample size	The sample size is visible in the graphs by individual data points corresponding to the individual animals. In other cases the exact sample size is stated in the figure legend. The number of mice was kept as small as possible with group sizes as accepted in the field with a minimum of n=3 for example in the FIB-SEM analysis (3D EM). Published examples are Djannatian et al., Nature Comm.(2019) 10:4794 https://doi.org/10.1038/s41467-019-12789-z, or Patzig et al. eLife (2016) 5:e17119. DOI: 10.7554/eLife.17119.<br>Exception: In Figure 2 (NanoSIMs) multiple measurements on only one animal are shown as a proof of principle. As shown in supplementary figure 7 these findings were reproduced in another animal.
Data exclusions	No data were excluded from the analysis. In Figure 1I-K in the proteome analysis proteins not detected were labeled n.d.
Replication	In Western blot analysis and RT-PCR in addition to biological replicates 3-4 technical replicated were used. Immunohistochemical staining was performed with 3 technical replicates in addition to 4-5 biological replicates. It is stated in Figure legends, in how many independent cohorts of mice of both genders the described findings were observed. All replication attempts were successful.
Randomization	Animals were kept in cages with mixed genotypes and allocated to groups by their age. For transmission electron microscopic analysis images were taken by systematic random sampling.
Blinding	Investigators were blinded to the genotype during data collection and analysis using animal numbers without genotype designation.

Reporting for specific materials, systems and methods

We require information from authors about some types of materials, experimental systems and methods used in many studies. Here, indicate whether each material, system or method listed is relevant to your study. If you are not sure if a list item applies to your research, read the appropriate section before selecting a response.

Materials & experimental systems

n/a	Involved in the study	
[ ]	[x]	Antibodies
[x]	[ ]	Eukaryotic cell lines
[x]	[ ]	Palaeontology and archaeology
[ ]	[x]	Animals and other organisms
[x]	[ ]	Human research participants
[x]	[ ]	Clinical data
[x]	[ ]	Dual use research of concern

Methods

n/a	Involved in the study	
[x]	[ ]	ChIP-seq
[x]	[ ]	Flow cytometry
[x]	[ ]	MRI-based neuroimaging

Antibodies

Antibodies used	Primary antibodies: APP 1:750 (Chemicon MAB348, RRID:AB_94882), CAII 1:1000 (gift from Said Ghandour (Ghandour et al., 1979));

	CD3 1:150 (Abcam ab11089, RRID:AB_369097), GFAP 1:200 (Novocastra NCL-GFAP-GA5, RRID:AB_563739), Iba1 1:200 (Abcam ab5076, RRID:AB_2224402), MAC3 1:500 (Pharmigen 553322, RRID:AB_394780); MBP 1:4000 (WB) 1:250 (IEM)(this study: for generation of MBP antisera, rabbits were immunized with the intracellular peptide 105-115 of the 21.5kDa isoform of mouse MBP (CQDENPVVHFFK). Anti-MBP antibodies were purified by affinity chromatography. The epitope is conserved in human and rat.), Olig2 1:100 (gift from Charles Stiles/John Alberta, DF308, (Sun et al., 2003)), Caspr1 1:500 (Neuromab K65/35, RRID:AB_2083496), Nav 1.6 1:500 (Alomone ASC-009, RRID:AB_2040202), PDGFRA 1:500 (Cell Signaling 3174, RRID:AB_2162345), PLP1 1:500 (polyclonal rabbit, A431 (Jung et al., 1996)), MAG 1:500 (Millipore Ab1567, RRID:AB_2137847), MOG 1:500 (gift from Christopher Linnington (Linnington et al., 1984)), Actin 1:5000 (Millipore Mab 1501, RRID:AB_2223041)<br>Secondary Antibodies for Immunoblotting:<br>HRP-goat anti-mouse IgG Dianova Cat# 115-035-003; 1:10000; HRP-goat anti-rabbit IgG Dianova Cat# 111-035-003; 1:10000<br>HRP-goat anti-rat IgG Dianova Cat# 112-035-167; 1:10000<br>Secondary Antibodies for Immunohistochemistry:<br>donkey-α-Mouse Alexa 555, Invitrogen Cat# A31570, 1:1000; goat-α-rabbit Dylight 633, Invitrogen Cat# 35562, 1:500
Validation	The MBP antibody was custom made and established for this study and successfully validated using shiverer mice lacking MBP as negative control. All other antibodies are either published and established or validated by the manufacturer.

Animals and other organisms

Policy information about studies involving animals; ARRIVE guidelines recommended for reporting animal research

Laboratory animals	Conditional MBP knock out was achieved by crossing MBP flox/flox mice with Mbp flox/flox::PLP-CreERT2+ mice (MGI:2663093) and kept on C57Bl6N background. The mice were housed in the mouse facility of the Max Planck Institute of Experimental Medicine with controlled ventilation (inward airflow, exhaust to the outside), temperature (21+/-2°C) and controlled rel. humidity (55% +/- 10%) in individually ventilated cages. Mice were group-housed 3-5 mice per cage in a 12-hour dark/light cycle with ad libitum access to food and water. All animals were treated with tamoxifen at an age of 8 weeks and analyzed at the ages of 24, 34, 48, 54 and 60 weeks as authorized by the by the Lower Saxony State Office for Consumer Protection and Food Safety (license 33.19-42502-04-16/2119) according to the German and European animal welfare laws. For the procedure of sacrificing vertebrates for subsequent preparation of tissue, all regulations given in the German animal welfare law (TierSchG §4) are followed. All procedures were supervised by the animal welfare officer and the animal welfare committee for the Max Planck Institute of Experimental Medicine, Göttingen, Germany. The animal facility at the Max Planck Institute of Experimental Medicine is registered according to §11 Abs. 1 TierSchG.
Wild animals	Study did not involve wild animals
Field-collected samples	Study did not involve field collected samples
Ethics oversight	All experiments with laboratory mice were performed in accordance with the German animal protection law.

Note that full information on the approval of the study protocol must also be provided in the manuscript.
